# Phrase Position, but not Lexical Status, Affects the Prosody of Noun/Verb Homophones

**DOI:** 10.3389/fpsyg.2018.01785

**Published:** 2018-09-25

**Authors:** Erin Conwell, Kellam Barta

**Affiliations:** ^1^Department of Psychology, North Dakota State University, Fargo, ND, United States; ^2^Department of English, North Dakota State University, Fargo, ND, United States

**Keywords:** lexical ambiguity, grammatical categories, lexical representation, speech production, prosody

## Abstract

Words that can occur in more than one lexical category produce regions of ambiguity that could confound language learning and processing. However, previous findings suggest that pronunciation of noun/verb homophones may, in fact, differ as a function of category of use, potentially mitigating that ambiguity. Whether these differences are part of the lexical representation of such words or mere by-products of sentence-level prosodic processes remains an open question, the answer to which is critical to resolving questions about the structure of the lexicon. In three studies, adult native speakers of English read aloud passages containing noun/verb homophones or nonce words used in both noun and verb contexts. Acoustic measurements of the target words indicated that, while sentence position influences the acoustic properties of noun/verb homophones, including duration and pitch, there are not significant effects of lexical category when other factors are controlled. Furthermore, the lexical status of a word (real or nonce) does not produce consistent prosodic effects. These findings suggest that previously reported prosodic differences in noun/verb homophones may result from the syntactic positions in which those categories tend to occur.

## Introduction

Ambiguity pervades natural language. Some of this ambiguity stems from words that have multiple meanings. These meanings may be related, as in the case of polysemes, or unrelated, as in the case of homophones. Some homophones and polysemes not only encode different meanings, but also belong to different grammatical categories (e.g., *run* may be used as both a noun and a verb). Such *ambicategorical* words could pose a significant challenge for language learning and processing, as they introduce both lexical and structural ambiguities into speech. However, children learn these words without apparent difficulty and adult speakers produce and process them every day, suggesting that the ambiguity they create is mitigated by other factors.

Although top-down factors such as syntax and plausibility of meaning no doubt play a role in resolving some of the ambiguities created by words that can be used in more than one grammatical category, prosodic and acoustic differences may provide low-level, bottom-up cues for rapidly classifying ambiguous words. A growing body of research indicates that words that are both nouns and verbs differ in their duration, pitch contours and/or vowel quality depending on the category in which they are used (Sorensen et al., 1978; Shi and Moisan, 2008; Li et al., 2010; Conwell and Morgan, 2012). Sorensen et al. (1978) reported that noun tokens of four English word types were reliably longer than verb tokens of the same words, but they also found that these effects disappeared when those words appeared in sentence-final positions. Analyses of spontaneous child-directed speech also find that nouns are reliably longer than verbs (Conwell and Morgan, 2012; Conwell, 2017). Further, English speakers systematically alter stress patterns in disyllabic nonce words depending on lexical class (Kelly and Bock, 1988; Kelly, 1992; Sereno and Jongman, 1995). Specifically, speakers tend to stress the first syllable of a nonce word when it is used as a noun and the second syllable when it is used as a verb. The grammatical category of a prime word also produces differential production of noun/verb homophones (Melinger and Koenig, 2007). These findings suggest that noun/verb homophones in English differ in pronunciation as a function of their category of use.

Studies have also found prosodic differences between noun and verb tokens of the same phonemic sequences in languages other than English. Canadian French speakers who read storybooks containing disyllabic nonce words aloud to infants lengthened the second vowels in noun uses of the nonce words more than they did in verb uses of the same word forms (Shi and Moisan, 2008). A similar study of Mandarin Chinese found that changes in duration and pitch across syllables distinguished noun and verb uses of disyllabic non-words in child-directed speech (Li et al., 2010). However, these studies are hard to generalize to English, as a significant proportion of category-ambiguous words in English are monosyllabic (Conwell and Morgan, 2012).

An additional question regarding possible differences in noun/verb homophone pronunciation is where such differences might originate. One possibility is that they may arise purely from phrasal prosody, as suggested by Sorensen et al. (1978). English exhibits strong phrase-final lengthening, in which items that occur at the end of constituent phrases have greater duration than those that appear in the middle of phrases (Cooper and Paccia-Cooper, 1980; Shattuck-Hufnagel and Turk, 1996). Nouns are more likely than verbs to appear in sentence-final and phrase-final positions, and thus they are more likely to be lengthened. Speakers also place prosodic boundaries after nouns more often than they place them after verbs (Watson et al., 2006), which increases the likelihood of nouns being lengthened relative to verbs. Additionally, phrase-final words are more likely to receive focal stress under most conditions than are phrase-medial words (Truckenbrodt, 2007) and nouns in some contexts necessitate focal stress, while verbs in similar contexts may not (Selkirk, 2011). Taken together, these facts suggest that differences in the duration of noun/verb homophones are simply products of prosodic processes that differentially affect nouns and verbs.

An alternative possibility is that pronunciation differences are associated with the specific meanings of the noun form and the verb form of the words themselves. A growing number of studies of homophones report representation-level differences in duration and pitch based on factors such as frequency and emotional content. Gahl (2008) found differences in the duration of homophone uses as a function of the frequency of the target meaning, suggesting that frequency of meaning, not frequency of the particular phonological sequence, impacts the phonetic realization of homophones. Nygaard et al. (2002) found that participants’ pronunciation of homophones was affected by the emotional content of the intended meaning. For example, the word *bridal* was produced with higher fundamental frequency (i.e., more positive emotion) than the word *bridle*. Melinger and Koenig (2007) demonstrated that the category of a prime affects which pronunciation of an ambiguous target word speakers produce. These findings indicate that lemma-level factors such as frequency, grammatical category, and meaning affect the pronunciation of homophonous words. Phonetic information that is specific to grammatical category could be stored as part of the lexical representation. Although Melinger and Koenig (2007) showed such effects for noun/verb pairs with phonemically distinct representations (e.g., *dove*) and for pairs with stress patterns that change as a function of category (e.g., *contrast*), there is not currently evidence for such effects in noun/verb pairs that are phonemically identical and monosyllabic, in other words, those that are truly homophonous. If there are differences in pronunciation as a function of lexical category, these differences could result from stored differences rather than from prosodic processes alone. If category-based pronunciation differences are stored in lexical representations, those differences could facilitate processing and learning of noun/verb homophones by providing a bottom-up cue to the intended category that is available to listeners before more top-down cues such as plausibility and syntax. This would facilitate processing of category-ambiguous words.

This article has two main goals. First, the previous work on the existence of acoustic differences in noun/verb homophones has notable limitations. Sorensen et al. (1978) obtained only one noun token and one verb token for each of four word types from each of their 10 participants, resulting in a very small sample. Because the focus of their research was speech timing, they measured only duration, not other cues, such as pitch. Conwell and Morgan (2012) and Conwell (2017) both examined child-directed speech, which differs prosodically from adult-directed speech (Ferguson, 1964), and might, therefore, not be generalizable. Therefore, one goal of the research presented here is to assess whether previously reported prosodic differences in ambicategorical words are robust across a range of tokens, speakers and sentence positions.

A second goal of this research is to begin to determine whether differences in noun/verb homophones are entirely the result of sentence prosody or whether they might be part of the lexical representation itself. This research addresses this issue in two ways. First, it controls for sentence and phrase position of the target words. Controlling sentence position reduces the effects of sentence prosody on pronunciation, but it does not wholly resolve the question of whether noun/verb differences are stored in the lexicon. Therefore, this work also compares the effects of grammatical category and sentence position on the realization of both real and nonce words used in noun and verb positions. Because nonce words have no lexical representation, any differences between the real and nonce words may be attributed to representational properties of the real words.

This article presents three studies intended to address these issues. In all three studies, adult native speakers of English read aloud passages containing noun/verb homophones in medial and final positions, while other participants read the same passages but with the noun/verb homophones replaced by nonce words. The target words were extracted and measured along a range of acoustic dimensions. Experiment 1 controlled for the sentence position of the target words, but not for phrase position of sentence-medial tokens. Experiment 2 compared phrase-medial tokens to sentence-final tokens of the target words, while Experiment 3 compared phrase-medial and phrase-final tokens. If pronunciation differences in noun/verb homophones are robust across speakers, we would expect noun and verb uses of the target words to differ significantly from one another as a function of category. If noun/verb homophone pronunciation is not affected by category of use, we would expect only to see pronunciation differences as a function of sentence position. Additionally, if the pronunciation distinctions between noun and verb uses of homophones are part of the lexical representations of these words, then we would expect no such differences in noun and verb uses of nonce words, as such words do not have lexical representations. Alternatively, any differences between noun and verb uses of real words could be entirely the result of post-lexical production processes, such as sentence prosody, in which case we would expect no effects of lexicality on those differences and the same patterns would be present in nonce words as well.

## Experiment 1

This experiment asks whether native English speakers reliably produce acoustic cues to nounhood or verbhood of ambicategorical words and how those cues might be related to sentence position and lexical status. Half of the participants read paragraphs aloud that contained noun/verb homophones in both sentence-medial and sentence-final positions. The other half read the same paragraphs with the noun/verb homophones replaced with nonce words. Analyses examined the role of lexical category, sentence position and lexical status on the pronunciation of these words. If differences in noun/verb homophones emerge as a function of sentence prosody alone, we would predict no differences in pronunciation as a function of category of use or of lexical status. If, however, a word’s category of use is related to its phonetic representation, independent of sentence position, then we would predict differences in pronunciation on the basis of lexical category for real words, but not for nonce words.

### Methods

#### Participants

Thirty-nine adult native speakers of American English, 21 male and 18 female, participated in this experiment for pay or for course credit. All participants were from the north-central United States and spoke the dialect of English associated with that region. Two additional participants were recorded, but their data were lost due to equipment failure. Participants were recruited from introductory psychology courses and by posting flyers around the North Dakota State University campus. This study was carried out in accordance with the recommendations of the NDSU Internal Review Board, which approved the experimental protocol. All participants gave written informed consent in accordance with the Declaration of Helsinki.

#### Procedure

Participants were seated approximately 12 inches from a Blue Snowball USB microphone connected to an HP laptop computer. Recordings were made using the software Audacity (Audacity, 2017). Participants read 72 paragraphs, each containing a target word. To facilitate fluency in reading, participants were encouraged to read all of the stimuli silently to themselves before recording commenced. Participants read at a conversational pace and volume. They were asked to monitor their own fluency and to repeat the entire paragraph if they stumbled or mispronounced a word. Most participants made such corrections fewer than three times during the study.

#### Stimuli

The real noun/verb homophones used in this study were *fish, kiss, kick, saw, walk, watch, match, nap*, and *pass*. These words were selected because both noun and verb uses exist in the child-directed speech of the Providence Corpus (Demuth et al., 2006). Each target word occurred eight times and each use was embedded in a 2–4 sentence paragraph, to reduce participants’ ability to identify the target words. Each target word occurred four times as a noun and four times as a verb. Sentence position was also manipulated such that each word appeared four times sentence-medially and four times sentence-finally. A complete list of stimuli for this experiment is provided in **Appendix A**. Sentence position and grammatical category were fully parameterized; each target word was used twice as a noun and twice as a verb in sentence-medial position as well as twice in each category in sentence-final position. Nineteen participants (9 male and 10 female) who read sentences containing real words were included in the analysis.

The nonce stimuli were identical to the stimuli that contained real words, except that the target words were replaced with *vik* (/vIk/), *blick* (/blIk/), *kip* (/kIp/), *gaw* (/gɔ/), *glot* (/glɔt/), *dotch* (/dɔtʃ/), *kasp* (/kæsp/), *lat* (/læt/), and *fash* (/fæʃ/). The mean phonological neighborhood density for these words is 22.7, according to the Irvine Phonotactic Online Dictionary (IPhOD; Vaden et al., 2009), compared to a mean phonological neighborhood density of 28.2 for the real words (Vaden et al., 2009). The neighborhood densities of the real and nonce words are not statistically different from one another [*t*(16) = 1.038, *p* = 0.315], indicating that any differences in the production of real and nonce words is not a product of different neighborhood densities. Participants practiced pronouncing the nonce words once before beginning the recording to ensure consistent productions across participants. Data from twenty participants (12 male and 8 female) who read nonce stimuli were analyzed.

#### Measurement

Trained research assistants listened to each recording and used Audacity to isolate the target word tokens. Using the speech analysis software PRAAT (Boersma and Weenink, 2017), boundaries were placed at the beginning and end of each target word and at the beginning and end of the vowels in those words. Boundary placement was based on auditory cues as well as on visual examination of the spectrogram and waveform. Token duration, vowel duration and mean pitch (in Hz) were automatically extracted. Pitch range was calculated in semitones, using the minimum and maximum pitch in the token.

A total of 42 targets (1.4%) were removed from the analysis, either because reading errors altered the lexical category or the position of the target word (7), because of disfluencies around the target word (8), or because the target word was mispronounced (27). Nine real targets and 33 nonce targets were excluded. Of the excluded nonce targets, 24 had been mispronounced. A total of 2766 tokens were included in the final analysis.

### Results

**FIGURE 1:**
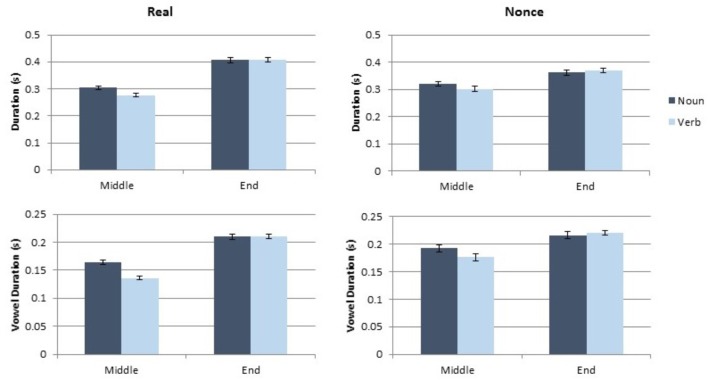
Results of the token and vowel duration measurements for Experiment 1.

**FIGURE 2:**
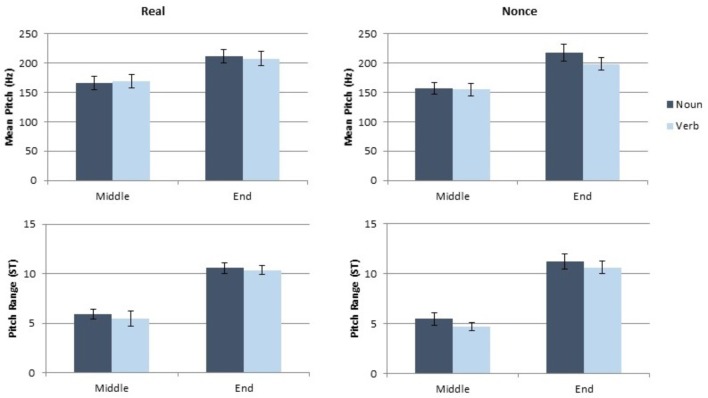
Results of the pitch and pitch range measurements for Experiment 1.

Each dependent measure (token duration, vowel duration, mean pitch and pitch range) was analyzed separately, using a linear mixed model implemented in R (R Core Team, 2015) using lme4 (Bates et al., 2015) and lmertest (Kuznetsova et al., 2017). Category (noun, verb), sentence position (medial, final), and lexical status (real, nonce), as well as the interaction of these variables, were included as fixed factors. Speaker gender (male, female) was also included as a fixed factor. Participant and paragraph were included as random effects. While a maximal random slopes structure is ideal for such analyses (Barr et al., 2013), every model of token duration in Experiment 1 that included random slopes failed to converge. Therefore, for appropriate comparison across experiments and measures, random slopes were not included in any model in this article. The token and vowel duration results from this study are presented in **Figure 1** and the mean pitch and pitch range results are presented in **Figure 2**.

#### Effects on Token and Vowel Duration

Category did not produce a significant main effect on token duration or on vowel duration (both *t* < 0.4, both *p* > 0.7). Sentence position did produce a significant main effect on token duration [*t*(189.11) = 2.17, *p* = 0.031] and a marginal main effect on vowel duration [*t*(180.32) = 1.86, *p* = 0.064]. Sentence-final tokens were longer and contained longer vowels than sentence-medial tokens did. Token duration also showed a marginal main effect of lexical status [*t*(215.91) = 1.95, *p* = 0.053], with real words showing longer duration than nonce words. The two-way of sentence position and lexical status was also statistically significant for token duration [*t*(209.36) = 2.42, *p* = 0.016], which results from a larger difference between sentence-medial and sentence-final tokens in real words than in nonce words. No other interaction of factors was statistically significant for token or vowel duration (all *t* < 1.4, all *p* > 0.17). Gender did not have a significant effect on token or vowel duration (both *t* < 0.7, both *p* > 0.5). Complete results from these analyses are presented in **Table 1**.

**Table 1:** Results of linear mixed model analysis of durational variables in Experiment 1.

Effect	Parameter	Token duration		Vowel duration	
			
		Estimate	*SE*	Estimate	*SE*
*Fixed effects*					
Intercept	β	0.361***	0.017	0.219***	0.011
Category	β	0.007	0.019	0.004	0.013
Sentence position	β	-0.042*	0.019	-0.024	0.013
Lexical status	β	0.04	0.021	-0.008	0.014
Gender	β	0.003	0.011	-0.004	0.007
Category × Position	β	-0.027	0.027	-0.021	0.018
Category × Status	β	-0.006	0.024	-0.002	0.016
Position × Status	β	-0.06*	0.024	-0.022	0.016
Category × Position × Status	β	-0.0003	0.035	-0.007	0.023
*Random effects*					
Participant	σ^2^	0.003	0.004	0.001	0.038
Item	σ^2^	0.001	0.002	0.0004	0.021
Residual	σ^2^	0.003	0.004	0.001	0.034


*Statistical significance was based on degrees of freedom estimated using the Satterthwaite approximation (^∗^p < 0.05, ^∗∗∗^p < 0.001).*

#### Effects on Pitch and Pitch Range

Category produced a marginal main effect on mean pitch [*t*(137.08) = 1.89, *p* = 0.061], with nouns exhibiting higher mean pitch than verbs. Mean pitch was also significantly affected by sentence position [*t*(135.02) = 6.04, *p* < 0.001] with higher mean pitch in sentence final tokens, but there was not a significant effect of lexical status on mean pitch [*t*(107.18) = 107.18, *p* = 0.26]. Mean pitch was further affected by speaker gender [*t*(38.82) = 5.88, *p* < 0.001], as male participants had lower mean pitch than female participants. No interaction of factors was statistically significant for mean pitch (all *t* < 1.4, all *p* > 0.18). Pitch range was significantly affected by sentence position [*t*(132.8) = 5.6, *p* < 0.001]. Final tokens had greater pitch range than medial tokens. Gender also significantly affected pitch range [*t*(39.69) = 2.89, *p* = 0.006] with male participants producing a smaller pitch range than female participants. The main effects of category and lexical status were not significant for pitch range, and no interaction of factors had a significant impact on pitch range (all *t* < 1, all *p* > 0.35). The complete results of the model of pitch and the model of pitch range are presented in **Table 2**.

**Table 2:** Results of linear mixed model analysis of pitch variables in Experiment 1.

Effect	Parameter	Mean pitch		Pitch range	
			
		Estimate	*SE*	Estimate	*SE*
*Fixed effects*					
Intercept	β	252.48***	11.43	11.5***	0.716
Category	β	-18.85	9.99	-0.282	0.844
Sentence position	β	-60.16***	9.95	-4.7***	0.839
Lexical status	β	-15.35	13.67	0.383	0.915
Gender	β	-59.34***	10.1	-1.433**	0.495
Category × Position	β	14.58	14.11	-0.202	1.19
Category × Status	β	18.4	13.8	-0.316	1.181
Position × Status	β	15.9	13.72	-1.083	1.172
Category × Position × Status	β	-10.92	19.38	0.082	1.657
*Random effects*					
Participant	σ^2^	499	22.34	2.28	1.51
Item	σ^2^	864.1	29.4	1.22	1.11
Residual	σ^2^	7217.9	84.96	74.64	8.64


*Statistical significance was based on degrees of freedom estimated using the Satterthwaite approximation (^∗∗^p < 0.01, ^∗∗∗^p < 0.001).*

### Interim Discussion

These findings suggest that lexical category does not affect the prosody of noun/verb homophones when sentence position is controlled for. This replicates the findings of Sorensen et al. (1978) with a larger number of participants and more word types. The positional effects on duration and pitch are consistent with English utterance-final prosody, which includes lengthening and increased pitch and pitch range (Shattuck-Hufnagel and Turk, 1996). Finding such effects suggests that participants were reading the stimuli in a naturalistic manner with sentence-level prosody that was typical for English. The effect of gender on mean pitch was also expected, although the smaller pitch range for male participants was somewhat unexpected. The effects of lexical status on token duration, specifically that nonce tokens had shorter duration than real tokens, and that real tokens show larger effects of sentence position than nonce tokens do, may indicate that participants were less natural in their production of sentences containing nonce words than those with real words. However, because the lexical status manipulation was a between-subjects factor, it is also possible that the effects of lexical status are a product of between-subjects variability in participants’ speech patterns. To fully match prosodic environments for the real and nonce words, it was necessary to use identical sentence contexts for those words. Having participants read the same sentences twice, once with a real word and once with a nonce word, would create demand characteristics that might affect pronunciation. Therefore, it was impractical to treat lexical status as a within-subjects factor.

The design of the stimuli in this experiment has two potential problems. First, although the sentence positions of the target words were parameterized, the phrase positions were not. Furthermore, differences in phrasal position could create differences in how likely a word is to be stressed or accented (Kratzer and Selkirk, 2007; Truckenbrodt, 2007). The second concern is that the specific vowels in the words used in this study are not particularly representative of the vowels in English. The target words were chosen on the basis of their frequency to ensure participants’ experience with both noun and verb forms, but not all of the vowels in the target words are frequent relative to other vowels. Specifically, /ɔ/ is the fifth least frequent vowel in American English (Kessler and Treiman, 1997). Frequent vowels may undergo reduction differently than less frequent vowels, which may have affected the vowel duration results.

To address these issues, we redesigned our stimuli to contain more representative vowels and to better control for phrase position. Experiment 2 directly controls for phrase position, allowing us to examine further how phrasal prosody might affect the production of noun/verb homophones. Sorensen et al. (1978) found that noun/verb homophones in phrase-final position did not exhibit durational differences as a function of category, but the strong tendency toward elongation in phrase-final positions could mask differences between noun and verb tokens. Sorensen et al. (1978) did not examine phrase-medial tokens.

## Experiment 2

This experiment is intended to clarify two issues regarding Experiment 1. First, it changes the target words to include vowels that are more frequent in English. Second, it controls for phrase position as well as sentence position. In Experiment 1, sentence-medial target words could appear anywhere in a clause or phrase, meaning that phrase position was not controlled for. In Experiment 2, all sentence-medial targets appear exactly two syllables before a phrase boundary. This manipulation will determine whether the lack of category effects in Experiment 1 are, in fact, due to a confound between category and phrase position that was present in the stimuli for Experiment 1.

### Methods

#### Participants

Thirty-eight adult native speakers of American English, 21 male and 17 female, participated in this experiment for course credit. All participants were recruited from undergraduate psychology courses at North Dakota State University and all spoke the regional dialect of English. This study was carried out in accordance with the recommendations of the NDSU Internal Review Board, which approved the experimental protocol. All participants gave written informed consent in accordance with the Declaration of Helsinki.

#### Procedure

The procedure for this experiment was identical to that in Experiment 1.

#### Stimuli

New stimuli were created for this experiment. The real noun/verb homophones used in this study were *test, check, pet, peel, lead* (/lid/), *beat, bit, clip*, and *kick*. These words were selected because they are widely used as both nouns and verbs and because they contain the high frequency vowels /ε/, /i/, and /I/. The average neighborhood density of these words was 34.8, according to IPhOD counts (Vaden et al., 2009). As in Experiment 1, each target word occurred eight times and each use was embedded in a 2–4 sentence paragraph, to reduce participants’ ability to identify the target words. Position and grammatical category were parameterized as in Experiment 1. All sentence-medial productions were exactly two syllables before the end of a syntactic phrase. The complete stimulus list for this study is in **Appendix B**. Twenty participants (11 male and 9 female) who read sentences containing real words were included in the analysis.

The nonce stimuli were identical to the stimuli that contained real words, except that the target words were replaced with *kesk* (/kεsk/), *pell* (/pεl/), *stek* (/stεk/), *deek* (/dik/), *keet* (/kit/), *neep* (/nip/), *bip* (/bIp/), *plick* (/plIk/), and *tib* (/tIb/). Based on IPhOD counts (Vaden et al., 2009), the average neighborhood density for these words is 24.2. The difference in neighborhood density is marginally significant [*t*(16) = 2.029, *p* = 0.059]. Participants practiced pronouncing the nonce words once before beginning the recording. Data from 18 participants (10 male and 8 female) who read nonce stimuli were analyzed.

A total of 107 targets (3.8%) were removed from the analysis, either because reading errors altered the lexical category or the position of the target word (14), because of disfluencies around the target word (22) or because the target word was mispronounced (71). Thirty-seven real targets and 75 nonce targets were excluded. Of the excluded nonce targets, 55 had been mispronounced, typically by changing the vowel (34 tokens) or using a real word instead (12 tokens). A total of 2736 tokens were included in the final analysis.

The target words were isolated and analyzed using the same process as in Experiment 1. The same four dimensions (token duration, vowel duration, mean pitch, and pitch range) were measured in the same way.

### Results

**FIGURE 3:**
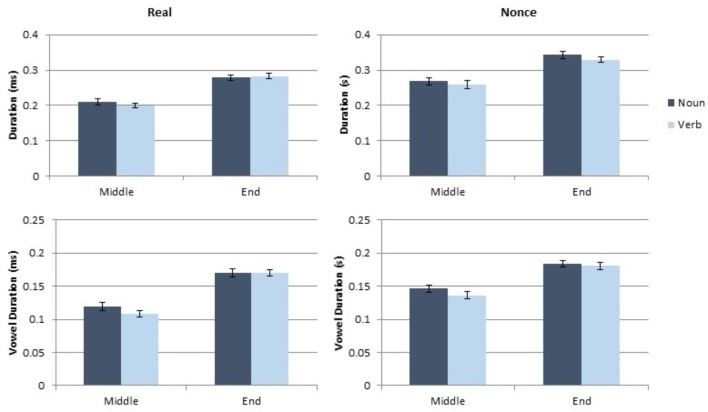
Results of token and vowel duration measurements for Experiment 2.

**FIGURE 4:**
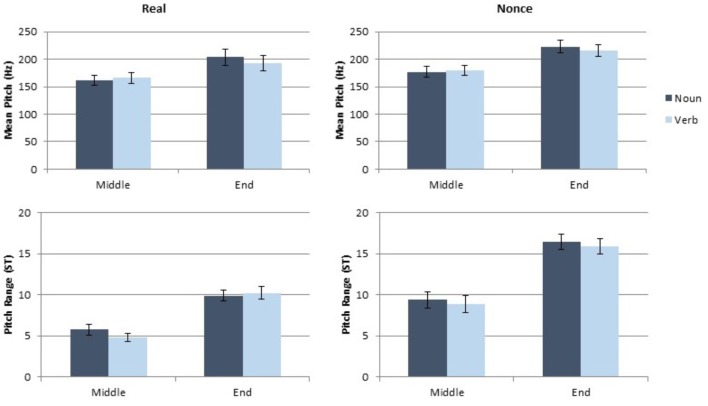
Results of pitch and pitch range measurements for Experiment 2.

The analyses for this experiment were conducted using the same process as in Experiment 1. Each dependent measure (token duration, vowel duration, mean pitch and pitch range) was analyzed separately, using a linear mixed model implemented in R (R Core Team, 2015) using lme4 and lmerTest (Bates et al., 2015; Kuznetsova et al., 2017). Category (noun, verb), sentence position (medial, final), and lexical status (real, nonce), as well as the interaction of these variables, were included as fixed factors. Speaker gender (male, female) was also included as a fixed factor. Participant and paragraph were included as random effects. The duration results of this study are presented in **Figure 3** and the pitch results are in **Figure 4**.

#### Effects on Token and Vowel Duration

As in Experiment 1, category produced no main effect on token duration [*t*(143.48) = 0.67, *p* = 0.51] or on vowel duration [*t*(143.8) = 0.44, *p* = 0.66]. Lexical status significantly affected token duration [*t*(165.13) = 3.2, *p* = 0.002]; real words were shorter than nonce words. However, lexical status did not affect vowel duration [*t*(162.62) = 1.24, *p* = 0.22]. Sentence position created significant main effects on token duration [*t*(143.47) = 4.27, *p* < 0.001] and vowel duration [*t*(143.79) = 3.95, *p* < 0.001]. Sentence-final tokens were longer and contained longer vowels than phrase-medial tokens did. Gender had no main effect on either durational measure (both *t* < 1.35, both *p* > 0.18). No interaction of factors significantly affected either token duration or vowel duration (all *t* < 1, all *p* > 0.3). The complete results of the models of token duration and vowel duration are presented in **Table 3**.

**Table 3:** Results of linear mixed model analysis of durational variables in Experiment 2.

Effect	Parameter	Token duration		Vowel duration	
			
		Estimate	*SE*	Estimate	*SE*
*Fixed effects*					
Intercept	β	0.352***	0.016	0.189***	0.009
Category	β	-0.011	0.017	-0.004	0.01
Sentence position	β	-0.073***	0.017	-0.038***	0.01
Lexical status	β	-0.066**	0.021	-0.014	0.012
Gender	β	-0.016	0.012	-0.008	0.007
Category × Position	β	0.002	0.024	-0.005	0.014
Category × Status	β	0.016	0.024	0.004	0.014
Position × Status	β	0.003	0.024	-0.013	0.014
Category × Position × Status	β	-0.016	0.034	-0.005	0.019
*Random effects*					
Participant	σ^2^	0.002	0.05	0.001	0.028
Item	σ^2^	0.001	0.035	0.0004	0.02
Residual	σ^2^	0.002	0.044	0.001	0.028


*Statistical significance was based on degrees of freedom estimated using the Satterthwaite approximation (^∗∗^p < 0.01, ^∗∗∗^p < 0.001).*

#### Effects on Pitch and Pitch Range

Mean pitch was significantly affected by sentence position [*t*(144.42) = 0.51, *p* < 0.001] and by gender [*t*(37.97) = 8.64, *p* < 0.001]. Sentence medial tokens had lower pitch than sentence final tokens and male participants had lower pitch than female participants. Neither category nor lexical status significantly affected mean pitch (both *t* < 1.5, *p* > 0.13). No interaction of factors significantly affected mean pitch (all *t* < 0.6, all *p* > 0.58). Pitch range was significantly affected by lexical status [*t*(114.7) = 5.2, *p* < 0.001]; real words had a smaller pitch range than nonce words. Sentence position also significantly affected pitch range [*t*(148.15) = 7.07, *p* < 0.001] with sentence final tokens showing a larger pitch range than sentence medial tokens. Neither category nor gender had a significant effect on pitch range (both *t* < 1.2, both *p* > 0.27). The analysis of pitch range further showed a significant interaction of lexical status and sentence position [*t*(140.89) = 2.16, *p* = 0.03] because nonce words showed a larger difference in pitch range between medial and final tokens than did real words. No other interaction of factors had a significant effect on pitch range (all *t* < 0.6, all *p* > 0.56). The complete results of the pitch and pitch range models are presented in **Table 4**.

**Table 4:** Results of linear mixed model analysis of pitch variables in Experiment 2.

Effect	Parameter	Mean pitch		Pitch range	
			
		Estimate	*SE*	Estimate	*SE*
*Fixed effects*					
Intercept	β	262.97***	10.56	15.966***	1.059
Category	β	-5.61	11.02	-0.499	0.999
Sentence position	β	-47.1***	11	-7.043***	0.997
Lexical status	β	-19.83	13.31	-6.723***	1.292
Gender	β	-70.21***	8.13	1.014	0.907
Category × Position	β	8.44	15.56	-0.101	1.41
Category × Status	β	-6.04	15.48	0.811	1.4
Position × Status	β	4.24	15.45	3.001*	1.392
Category × Position × Status	β	8.46	21.86	-1.14	1.97
*Random effects*					
Participant	σ^2^	796.4	28.22	4.57	2.14
Item	σ^2^	547	23.39	6.64	2.58
Residual	σ^2^	4909.8	70.07	73.39	8.57


*Statistical significance was based on degrees of freedom estimated using the Satterthwaite approximation (^∗^p < 0.05, ^∗∗∗^p < 0.001).*

### Interim Discussion

The goal of Experiment 2 was to determine two things: whether the findings from Experiment 1 would generalize to more representative vowels and whether differences in phrasal position could account for the lack of effects of lexical category. However, like Experiment 1, Experiment 2 showed no effects of category on any prosodic measure. Furthermore, the effect of lexical status on token duration was reversed in this experiment. Once again, the most consistent effects on all measures were those of sentence position, not category or lexical status. However, in controlling for phrasal position of sentence-medial words, Experiment 2 actually contrasted phrase-medial position with sentence-final position. It does not clarify whether there are effects of phrase-medial and phrase-final position in sentence-medial contexts. To resolve this issue, the stimuli were slightly revised for an additional experiment.

## Experiment 3

This experiment uses the same target words as Experiment 2, but modifies the stimuli so that the positional contrasts are between phrase-medial and phrase-final tokens. This manipulation will help to elucidate the lack of lexical category effects seen in Experiments 1 and 2.

### Methods

#### Participants

Forty-one adult native speakers of American English (9 male, 31 female, and one who declined to indicate sex or gender) participated in this experiment for course credit. All participants were recruited from undergraduate psychology courses at North Dakota State University and all spoke the regional dialect of English. Two additional participants completed the study but were excluded due to strong non-regional accents that affected their vowel productions. Another four participants completed the study and were excluded from analysis because of high rates of mispronunciations of target words (>15%) or significant disfluencies throughout the study. This study was carried out in accordance with the recommendations of the NDSU Internal Review Board, which approved the experimental protocol. All participants gave written informed consent in accordance with the Declaration of Helsinki.

#### Procedure

The procedure for this experiment was identical to that of Experiments 1 and 2.

#### Stimuli

The stimuli for this study were slightly modified versions of those used in Experiment 2. The sentences with phrase-medial targets were identical to the stimuli for Experiment 2. The sentences with targets in final position were modified so that the target words were sentence-medial, but phrase-final. A complete list of stimuli for this experiment can be found in **Appendix C**. Twenty participants (5 male and 15 female) who read sentences containing real words and 21 (4 male, 16 female and 1 unindicated) who read sentences containing nonce words were included in the analysis.

A total of 83 targets (2.8%) were removed from the analysis, either because reading errors altered the lexical category or the position of the target word (14), because of disfluencies around the target word (35) or because the target word was mispronounced (34). Twenty-eight real targets and 55 nonce targets were excluded. Of the excluded nonce targets, 25 had been mispronounced, typically by changing the vowel (14 tokens) or using a real word instead (10 tokens). A total of 2869 tokens were included in the final analysis.

The target words were isolated and analyzed using the same process as in Experiments 1 and 2. The same four dimensions (token duration, vowel duration, mean pitch, and pitch range) were measured in the same way.

### Results

**FIGURE 5:**
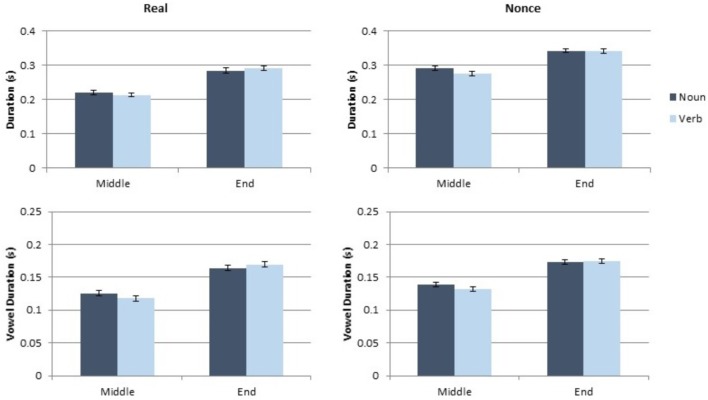
Results of token and vowel duration measurements for Experiment 3.

**FIGURE 6:**
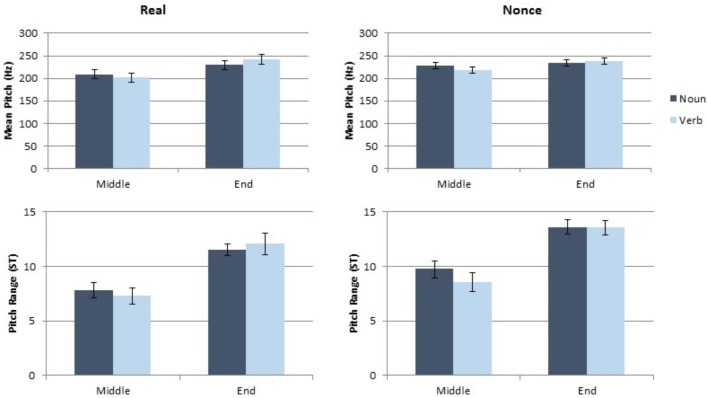
Results of pitch and pitch range measurements for Experiment 3.

The data were analyzed in the same manner as the data for Experiments 1 and 2. Each dependent measure (token duration, vowel duration, mean pitch, and pitch range) was analyzed separately, using a linear mixed model implemented in R (R Core Team, 2015) using lme4 and lmerTest (Bates et al., 2015; Kuznetsova et al., 2017). Category (noun, verb), sentence position (medial, final), and lexical status (real, nonce), as well as the interaction of these variables, were included as fixed factors. Speaker gender (male, female) was also included as a fixed factor. Participant and paragraph were included as random effects. The duration results of this study are presented in **Figure 5** and the pitch results are in **Figure 6**.

#### Effects on Token and Vowel Duration

As in Experiments 1 and 2, grammatical category did not produce significant main effects on token duration [*t*(155.99) = 0.22, *p* = 0.82] or vowel duration [*t*(151.5) = 0.57, *p* = 0.57]. Lexical status significantly affected token duration [*t*(192.56) = 3.14, *p* = 0.002], but not vowel duration [*t*(183.8) = 0.52, *p* = 0.61]. In this case, real words were shorter than nonce words. Phrase position also showed a significant main effect for token duration [*t*(215.17) = 3.03, *p* = 0.003], as well as for vowel duration [*t*(186.5) = 2.87, *p* = 0.004]. Phrase-final tokens were longer and contained longer vowels than phrase-medial tokens did. No interaction of factors produced a significant effect on either of the durational measures (all *t* < 1.39, all *p* > 0.18). The complete results of the models for token duration and vowel duration are presented in **Table 5**.

**Table 5:** Results of linear mixed model analysis of durational variables in Experiment 3.

Effect	Parameter	Token duration		Vowel duration	
			
		Estimate	*SE*	Estimate	*SE*
*Fixed effects*					
Intercept	β	0.34***	0.012	0.001***	0.002
Category	β	0.004	0.016	0.0002	0.001
Sentence position	β	-0.045**	0.015	0.001**	0.003
Lexical status	β	-0.055**	0.017	0.001	0.002
Gender	β	-0.003	0.008	0.0001	0.001
Category × Position	β	-0.023	0.022	0.001	0.003
Category × Status	β	0.002	0.023	0.001	0.002
Position × Status	β	-0.02	0.022	0.0002	0.001
Category × Position × Status	β	0.011	0.032	0.0012	0.003
*Random effects*					
Participant	σ^2^	0.002	0.048	0.001	0.028
Item	σ^2^	0.001	0.023	0.0002	0.013
Residual	σ^2^	0.002	0.047	0.001	0.035


*Statistical significance was based on degrees of freedom estimated using the Satterthwaite approximation (^∗∗^p < 0.01, ^∗∗∗^p < 0.001).*

#### Effects on Pitch and Pitch Range

Only speaker gender had a significant main effect on mean pitch [*t*(41.03) = 3.57, *p* = 0.001] with male participants exhibiting lower pitch than female participants. No other factor or interaction of factors had a significant effect on mean pitch (all *t* < 1.02, all *p* > 0.31). Pitch range was also significantly affected by gender [*t*(40.99) = 2.67, *p* = 0.011]; male participants showed greater pitch range over the target words than female participants did. Pitch range was marginally affected by lexical status [*t*(116.66) = 1.73, *p* = 0.086] with real words containing a smaller pitch range than nonce words. Pitch range was also significantly affected by phrase position [*t*(144.31) = 4.17, *p* < 0.001]. Phrase final words had greater pitch range than phrase medial words did. Grammatical category did not produce a significant main effect on pitch range [*t*(140.57) = 0.34, *p* = 0.97]. No interaction of factors produced a significant effect on pitch range (all *t* < 1.16, all *p* > 0.25). The complete results of the models for pitch and pitch range are presented in **Table 6**.

**Table 6:** Results of linear mixed model analysis of pitch variables in Experiment 3.

Effect	Parameter	Mean pitch		Pitch range	
			
		Estimate	*SE*	Estimate	*SE*
*Fixed effects*					
Intercept	β	242.38***	9.98	12.89***	0.83
Category	β	5.06	11.23	0.03	0.86
Sentence position	β	-5.33	11.1	-3.59***	0.86
Lexical status	β	-2.37	13.82	-1.99	1.15
Gender	β	-35.48***	9.95	2.54*	0.95
Category × Position	β	-15.12	15.79	-1.41	1.22
Category × Status	β	7.12	15.96	0.58	1.23
Position × Status	β	-16.11	15.86	-0.09	1.23
Category × Position × Status	β	-5.35	22.5	0.24	1.74
*Random effects*					
Participant	σ^2^	823.3	28.69	2.96	1.72
Item	σ^2^	655.4	25.6	5.7	2.39
Residual	σ^2^	6336.1	79.6	75.88	8.71


*Statistical significance was based on degrees of freedom estimated using the Satterthwaite approximation (^∗^p < 0.05, ^∗∗∗^p < 0.001).*

## General Discussion

The studies presented here asked two questions. First, they examined whether there are reliable differences between noun and verb uses of homophones in English. They also asked how factors such as lexical status and position within an utterance would affect the phonetic realization of words with more than one grammatical category. The use of nonce words, which have no stored representation for our participants, was intended to allow discussion of the differences in noun/verb homophones that are and are not part of the lexicon, while controlling for phrase and sentence position. The results of these three studies are consistent in some ways, but deviate from one another in others. A summary of results from all three studies can be found in **Table 7**.

**Table 7:** Summary of *t*-values across three experiments.

	Token duration	Vowel duration	Pitch	Pitch range
**Category (noun)**				
Experiment 1	0.37	0.31	-1.89	-0.34
Experiment 2	-0.67	-0.44	-0.51	-0.5
Experiment 3	0.22	0.57	0.45	0.03
**Position (middle)**				
Experiment 1	-2.17*	-1.863	-6.04***	-5.6***
Experiment 2	-4.27***	-3.95***	-4.28***	-7.07***
Experiment 3	-3.03**	-2.871**	-0.48	-4.17***
**Lexical Status (real)**				
Experiment 1	1.95	-0.59	-1.12	0.42
Experiment 2	-3.2**	-1.24	-1.49	-5.2***
Experiment 3	-3.14**	-0.52	-0.17	-1.73
**Gender (male)**				
Experiment 1	0.31	-0.62	-5.88***	-2.89**
Experiment 2	-1.34	-1.25	-8.64***	1.12
Experiment 3	-0.4	-1.34	-3.57***	2.67*
**Category (noun)^∗^Position (middle)**				
Experiment 1	-1	-1.16	1.03	-0.17
Experiment 2	0.072	-0.36	0.54	-0.07
Experiment 3	-1.04	-1.34	-0.96	-1.15
**Category (noun)^∗^Lexical Status (real)**				
Experiment 1	-0.25	-0.11	1.33	-0.27
Experiment 2	0.65	0.32	-0.39	0.58
Experiment 3	0.1	0.003	0.45	0.47
**Position (middle)^∗^Lexical Status (real)**				
Experiment 1	-2.42*	-1.36	1.16	-0.92
Experiment 2	0.12	-0.96	0.27	2.16*
Experiment 3	-0.91	-0.94	-1.02	-0.08
**Category^∗^Position^∗^Lexical Status**				
Experiment 1	-0.01	-0.29	-0.56	0.05
Experiment 2	-0.47	-0.26	0.39	-0.58
Experiment 3	0.35	0.26	-0.24	0.14


**Statistical significance was based on degrees of freedom estimated using the Satterthwaite approximation (^∗^p < 0.05, ^∗∗^p < 0.01, ^∗∗∗^p < 0.001).**

Across three studies, we have failed to find evidence that nouns are consistently longer than verbs when other factors that influence prosody are considered. These results replicate those of Sorensen et al. (1978), while expanding the analysis to a greater number of word types and speakers, as well as accounting for multiple factors that might influence lexical prosody. We consistently find an effect of phrase or sentence position on the durational and pitch properties of these words. Because English is a head-final language, nouns are more likely than verbs to appear in final positions in spontaneous speech. In light of this fact, previous work that identifies category effects on token duration in spontaneous speech (e.g., Conwell and Morgan, 2012; Conwell, 2017) may find those effects due to a confound between lexical category and utterance positions, as well as the tendency of speakers to place prosodic boundaries more often after nouns than after verbs (Watson et al., 2006). Although previous work has statistically controlled for positional effects, direct experimental manipulation provides a more precise control. These results indicate that, once known production factors are well-controlled for, noun/verb homophones do not differ in their durations. This does not necessarily mean that such differences are unimportant for language learning and processing, as prosodic information may be available to speakers and learners before syntactic information is (or, alternatively, prosodic information may carry critical syntactic information). These results simply mean that the differences that have been previously reported may not be properties associated with “nounhood” or “verbhood,” *per se*. Rather, they tend to co-occur with grammatical category because of the interaction of prosody and syntax. This results in a correlation of lengthening with category, but not a causal relationship in which category causes lengthening or shortening (see Kratzer and Selkirk, 2007, for a related analysis of pitch accent and category).

The inconsistent effects of lexical status are difficult to interpret. The lexical status manipulation was intended to address whether the presence of a stored representation for a word affects the extent to which its category of use impacts is phonetic realization. However, as this research found no effects of grammatical category and inconsistent effects of lexical status (e.g., in Experiment 1, real word were longer than nonce words, but in Experiment 2, this effect was reversed), these data cannot be brought to bear on that question. Lexical status was manipulated as a between-subjects factor so we could control local phonetic context of the real and nonce words by presenting them in otherwise identical paragraphs. Unfortunately, this means that we were unable to account for participant differences in the lexical status analyses. With respect to the main question that this research was intended to address, however, lexical status did not interact with grammatical category of use, indicating that there are not differences in the production of real noun/verb homophones that are not present in the production of nonce words in those same contexts.

One factor that may have influenced the production of distinctions between noun and verb tokens of category ambiguous words in these studies is the relatedness of the noun and verb meanings. For some of the word types used in this study (e.g., *kick*, *peel*), the noun and verb forms are closely related. In the case of *kick*, for example, the noun use derives from the verb form without overt morphology. Other word types have wholly distinct meanings. For example, *saw* in its noun form refers to a hand tool, while *saw* in its verb form is the past tense of *see*. Other words have noun and verb meanings that may be historically related (e.g., *watch*), while still other pairs have both related and unrelated meanings (e.g., *pass*, *check*). It is possible that the extent to which the two forms are semantically related may affect how differentiated they are in production. Words with completely distinct meanings could be more differentiated than those that are more closely related, as, in some theories of lexical organization (e.g., Barner and Bale, 2002), words with more closely related meanings may share a lexical entry, while words with distinct meanings likely do not. However, previous work on spontaneous speech suggests that derived homophone pairs do differ in duration as a function of lexical category (Lohmann, 2017). An ideal test of this issue would include degree of meaning relatedness as a factor in the statistical analysis, but procedures for quantifying meaning relatedness of homographic words are not readily available. Therefore, these differences in relatedness cannot be statistically accounted for, which is a limitation of these results.

The only consistent effects across the three experiments in this study are patterns that have been widely reported in previous literature, namely, that syntactic position affects the prosody of words. These data contribute not only further evidence that words before syntactic boundaries are subject to lengthening and increased pitch and pitch range, but also evidence that, once such prosodic effects have been controlled for, previously reported effects of grammatical category on prosody are no longer seen. This suggests that those effects are less the result of stored prosodic differences between nouns and verbs than the result of production processes operating at the level of the sentence. Our consistent effects of position, both phrasal and sentential, on duration and pitch measures are consistent with the phrase and sentence prosody of English (Shattuck-Hufnagel and Turk, 1996) and with prior work on the relationship between prosodic and syntactic phrasing (Selkirk, 2011).

The research presented in this article shows that when a range of factors, including lexical status and position in a sentence, are controlled for, the previously reported effects of grammatical on the prosody of noun/verb homophones disappear. Furthermore, lexical status does not have a consistent effect on the prosody of such words and there are no interactions of category and lexical status, nor of category and position on these factors. These findings may indicate that low-level phonetic features of noun/verb homophones are entirely the result of production processes, including sentential prosody.

## Author Contributions

KB collected and analyzed data for Experiment 1, wrote the first draft of the method sections, and provided feedback on the other portions of the manuscript. EC designed the experiments and stimuli, analyzed the data for Experiments 1–3, wrote the introduction, results, and discussion sections, and edited the methods section. EC was responsible for the final edit of the manuscript.

## Conflict of Interest Statement

The authors declare that the research was conducted in the absence of any commercial or financial relationships that could be construed as a potential conflict of interest.

## APPENDIX A

### Complete Stimulus List for Experiment 1

It was Memorial Day today. Everyone who came to our cook-out loved the **fish/blick**. There were also some great salads.

As science progresses, ideas have to change. People used to think that whales were **fish/blick**. Of course, they had to rethink that once they learned that whales are really mammals.

It took me forever to figure out what I was going to make for supper. I love eating grilled **fish/blick** with fresh spinach. That’s what I decided to cook.

My family really enjoyed our trip to the aquarium. Some **fish/blick** are very colorful but others are plain. We were especially excited to see the sharks and the turtles.

My dad and I spend our weekends at the lake. Although it sometimes rains, we still **fish/blick**. It is one of my favorite hobbies.

The whole class was excited for the end of the year camping trip. After setting up their campsite, everyone decided it would be fun to **fish/blick**. No one caught anything, but they had a good time.

My dad wanted to take a trip to the mountains. I would rather **fish/blick** than hunt. He likes hunting better, though, so that’s what we did.

My friend Greg and I had a very long day at work today. We were both stressed and wanted to go out and have some fun. I said to him, “We should **fish/blick** tonight if you have time.”

David has a very nice **kick/kip**. I think that is one of the reasons why his team wins so much. They did great this season and are now heading to the championship game.

The star player was the hero of the game. He celebrated after his successful penalty **kick/kip**. His team didn’t win, but it was a great play.

The score had been close all through the game. With one perfect **kick/kip** through the uprights, the Vikings pulled ahead. Now all they had to do was make sure the Packers didn’t score.

Everyone at my school likes to make people laugh. My friend told the best joke ever. I got a **kick/kip** out of that hilarious joke.

The game was getting intense. Shelley had the ball, but there was no one open. Before another defender came, she knew she had to **kick/kip**.

Maggie and Aidan were really bored. The children spent a long time looking for a ball to **kick/kip**. They couldn’t find one, so they drew on the sidewalk with chalk instead.

We all wanted to play a game, but we have too many options. We need to make a decision. Let’s **kick/kip** the soccer ball.

George’s boss told him to re-do his entire project. He had been working on it for 6 months. He got so mad that he wanted to **kick/kip** the wall.

At the end of their seventh date, the girl was so excited when she got a **kiss/vik**. The boy was also excited.

The new grandmother was so excited. She picked up the baby and gave it a big **kiss/vik**. Then she cradled it and sang a lullaby.

This weekend, my friend got married. Her father gave her a **kiss/vik** on the cheek before he walked her down the aisle. Then, he sat down and the ceremony began.

The author knew exactly how to end her book. There’s something special about a **kiss/vik** under the stars, so the two main characters ended up sitting on the riverbank late at night.

Everyone gathered to see the royal couple **kiss/vik**. Thousands of people stood in front of the palace and many others turned on the TV. It was a huge day for the country.

The time had come. Angie and Mike were finally married. At the end of the wedding, they had to **kiss/vik**. Everyone cheered.

My sister was looking forward to next week. She got a free get-away because she attended so many conferences for work. She got in trouble so she can **kiss/vik** her vacation goodbye.

During the final season, many people were curious how the show would end. Fans wanted the main characters to **kiss/vik** in the finale. The writers refused to say whether that would happen.

At the wrestling tournament, there was a very good **match/lat**. The crowd was very excited. Finally, Patrick was declared the winner.

It was a very cold night. Once the fire was burning, Charlie blew out the **match/lat**. Having a fireplace is nice in the winter.

The barbeque was going well. Rose struck a **match/lat** against the steps to light the grill. Everyone waited patiently while the food cooked.

It was definitely a good **match/lat** at the basketball game last night. The two teams have a long-standing rivalry. A lot of people came, and a bunch of money was raised to help a charity.

Today in my fashion design class, I realized I had made a mistake. None of the colors were supposed to **match/lat**. I had to start my final project over again.

We need to redecorate the dining room. The curtains and the trim in that room don’t **match/lat**. It’s very unpleasant to eat in there.

My mother was helping me do laundry. She said, “Make sure that you **match/lat** the correct pair of socks.” I would be embarrassed if I wore two socks of different colors.

When universities **match/lat** roommates, they try to make everyone happy. Unfortunately, sometimes they have to make difficult decisions. Many students do not get along.

My birthday was yesterday. My friends took me out to eat, and then we went dancing. After last night’s celebration, I really need a **nap/kasp**.

We were trying to decide what to do after our **nap/kasp**. Some of us wanted to go shopping but others wanted a snack. We ended up doing both.

An hour-long **nap/kasp** helped Greta feel better. After she woke up, she was able to finish writing her paper.

My co-worker was gone today and I had to do all of his work. My boss was not understanding at all. I really needed a **nap/kasp** after work today.

My pets are very different. My cat loves to **nap/kasp**. My dog wants to run around all day.

Bobby had been at daycare playing all day. His parents finally got off work and took him home. He was so tired, but he still wouldn’t **nap/kasp**.

Bill took the crayons and colored all over the walls. He had to have a timeout because of his naughty behavior. It was time for him to **nap/kasp** after that.

Mark got the baby to **nap/kasp** and then finished cleaning the kitchen. After the baby woke up, they walked the dog to the park and took lots of pictures.

Because I went to so many baseball games, I received a special **pass/fash**. I love watching baseball. My friends play very well and the peanuts and hot dogs are delicious.

Kevin was a great hockey player. Suddenly trapped by two defenders, he made a quick **pass/fash**. That quick thinking helped his team win the game.

Greg attended a bunch of games last year. He has a free **pass/fash** into today’s football game. He hopes he’ll get to meet the quarterback afterwards.

The crowd was very disappointed after the football game. The receiver dropped the **pass/fash** and made his team turn over the ball. The other team scored and won the game.

Since time was running out and he wasn’t close enough to shoot, Henry knew he had to **pass/fash**. Then the defender stole the ball and won the game. Everyone was disappointed.

The final for statistics was tomorrow. Even though Steve liked the class, he knew he had to study late if he was going to **pass/fash**. He also wanted to be sure to get a good night’s sleep.

My favorite basketball team is behind. The point guard should really **pass/fash** the ball to someone else. Maybe, they could start scoring then.

Lily hoped she would **pass/fash** her chemistry class but knew she would have to work hard. She went to her professor, who gave her some extra practice problems. She aced the final and did well in the class.

The scenery workshop for the play is tomorrow. I got a note after rehearsal. It said, “Please bring the following items tothe workshop: a wrench, a hammer, and a **saw/glot**.”

We were clearing out the backyard to make room for a patio. We had to get rid of an old, sick elm. The tree trunk was very thick, so we needed a good **saw/glot**.

My new **saw/glot** came in the mail 2 days ago. I have been waiting for it for about a month now. I am working on a present for my brother.

Cary and Pat were building a new deck. The circular **saw/glot** was very useful for that project. It helped them finish quickly.

The daylight bank robbery was very unusual. The witnesses had to tell the police what they **saw/glot**. Still, it was hard for them to identify the criminals.

Joe and Bob have been building a doghouse. After taking exact measurements of the wood, the next step was to **saw/glot**. They were very excited for this part of the project.

I haven’t seen my friend Kate in a long time. I thought I **saw/glot** her at the mall last week, but it was just someone who looked like her.

When I **saw/glot** that my neighbor was selling her house, I was very sad. She has two nice kids who help the older man next door with his yard. The neighborhood will miss them.

Sally had a very busy day. Before work, she had to do laundry. After work, she went to a movie. After that, she went for a **walk/gaw**. Finally, she went home and ate supper.

Sarah and Zach needed to go to the store. They decided not to drive because it was only a short **walk/gaw**. They didn’t buy much and it didn’t take them very long.

Barb had a very long day. She took a long **walk/gaw** after she got home from work. That helped her relax and feel better.

Because the weather was nice, the family decided not to drive. The **walk/gaw** from the library felt long because they were carrying so many books. Still, they were glad they hadn’t taken the car.

Barb lived a part of town where the busses didn’t pick up students. When she had to go to school, she had to **walk/gaw**. Fortunately, it wasn’t that far from school.

The 5k was held to raise money for childhood diabetes. Because it wasn’t a competitive race, participants could decide whether they would run or **walk/gaw**. Most people had a great time.

I like to **walk/gaw** to work, but not when it’s raining. I take the bus when the weather is bad and sometimes, I ask my co-worker for a ride.

Andy and Tasha wanted something to do. They decided to **walk/gaw** to the beach together. They enjoyed seeing the tide go out.

For Mother’s Day, the kids decided to get their mom some new jewelry. It took Darlene a while to decide whether she wanted the bracelet or the **watch/dotch**. She eventually decided on the bracelet.

I went to a brand-new store this weekend and bought a fancy **watch/dotch**. It had a bunch of gadgets on it. It tells me what time it is in London.

My favorite jewelry company is coming out with a new line. I like that their new product combines the precision of a Swiss **watch/dotch** with French style. I think I’ll buy one for my mother.

I just bought some gear for running. I got a brand new **watch/dotch** on Saturday. It has some really cool technology.

The museum is a very busy place. One guard had to go eat lunch. When she left, it was time for a new one to **watch/dotch**.

In the semi-final, Nate hurt his ankle after making a great save. Instead of playing in the championship, he had to sit and **watch/dotch**. He was glad to see his team win.

Mary could hardly **watch/dotch** as her favorite team lost the championship. They had been ahead at halftime, but lost the lead early in the fourth quarter.

When we go to the zoo, these things will happen. The gorilla will **watch/dotch** the monkeys eat their lunch. The bears will chase each other and the elephants will wash themselves.

## APPENDIX B

### Complete Stimulus List for Experiment 2

Even though Ronnie knew his mom was baking a pie for a bake sale, he still snuck in the kitchen and stole a **bit/tib**. It was cherry, which is his very favorite. He was in lots of trouble when his mom found out.

Rebecca noticed that no one had tried her casserole, not even a tiny **bit/tib**. She was disappointed because she had worked hard to get the recipe just right.

Eddy saw the delicious cake and took a **bit/tib** slyly. He hoped no one would notice that it was gone. The cake was for a party, but he just couldn’t wait that long.

I went to a potluck at my church this weekend. It was okay, but most of the dishes were very bland. I prefer food that has a **bit/tib** of spice, not just starch.

The tiger had been stalking the monkey through the jungle for several minutes. Then she saw her prey nearby and quickly **bit/tib**. The monkey never knew what happened.

When Fluffy got nervous, she hissed and **bit/tib**. The veterinarian was well prepared, though, and moved her hand out of the way quickly. Cats get scared very easily.

When she saw her owner, the dog was so excited that she ran in a circle and **bit/tib** her tail. Her owner had been away for several weeks and she was very happy to see him.

Oliver was chewing too fast and **bit/tib** his lip. He winced in pain and made a loud sound. Everyone turned to stare at him and he was very embarrassed.

I heard that the new quarterback also has an excellent **kick/bip**. That might make for some interesting plays this season. Football can be an unpredictable sport, so it’s important to have flexible skills.

When the soccer ball came toward Wayne, he got so nervous that he missed the **kick/bip**. He preferred math and science to sports because he’s better at them. His mom thought soccer would be a good way for him to meet people.

My karate teacher told me that I should work on my middle **kick/bip** for class. We’ll be fighting next month and I want to be prepared. Martial arts require a lot of discipline.

Frank just started playing soccer and he thinks his **kick/bip** is bad. He’s been practicing all afternoon and he’s starting to see a little improvement. If he works on it every day, he’ll be much better by the time they start playing games.

Max is a toddler, so when he gets frustrated all he knows how to do is scream and **kick/bip**. His mom and dad have been trying different techniques to avoid these tantrums. They haven’t found anything that works yet.

Tasha and Aidan were really bored. The children spent a long time looking for a ball to **kick/bip**. They couldn’t find one, so they drew on the sidewalk with chalk instead.

Dylan was so angry that he wanted to **kick/bip** a wall, but he restrained himself. He’s been working hard to be more constructive with his anger. He took a few deep breaths to calm down.

After the other team missed a pass, Lilly ran forward to **kick/bip** the ball, but she slipped on a patch of wet grass. She fell and twisted her ankle and had to leave the game. She was really disappointed because soccer is her favorite sport to play.

I went rock climbing for the first time last night. I felt very safe knowing that I was attached with the rope and the **clip/plick**. I had a great time and I’ll probably go again.

After finishing the movie, the director made a promotional **clip/plick**. She sent it to potential distributors with the hope that one of them would help her promote her film.

The teacher suggested that his students should use a paper **clip/plick** for drafts. That way, the pages won’t get disorganized, but they can be rearranged easily.

Anna realized she lost her pink hair **clip/plick** at school, so she went back to look for it. It had fallen under her desk. She was glad to find it because it was her favorite.

The special agent didn’t have much experience defusing bombs. He really hoped that he chose the right wire to **clip/plick**. When the bomb didn’t explode, he knew he had made the right choice.

Kathy spent all day looking for a pair of scissors. Her coat had a loose thread that she wanted to **clip/plick**. She finally found a pair in the supply closet at work.

Jennifer wanted to paint her nails but she had to **clip/plick** them first because she didn’t like keeping them long. She plays guitar and having long nails makes it hard to switch between chords.

After Jake was done mowing the lawn, he was supposed to **clip/plick** the hedge, but he got really tired and forgot. Mrs. Martin didn’t pay him as much as she had promised because he didn’t finish the job.

Even though Bobby had turned 16, he couldn’t drive until he passed the road **test/stek**. He did well on everything except parallel parking. Still, he did well enough to get his license.

Sarah finished studying around midnight and the next morning, she took her **test/stek**. She wished she had slept more because she was exhausted and couldn’t concentrate.

Micah never studies in advance. He took a chemistry **test/stek** Monday, even though he wasn’t prepared. Although he didn’t do well, he hopes to improve his grade on the next one.

Natalie was sorry that she had been sick on Friday. The teacher handed out the study guide for a hard **test/stek** that day. Everyone is worried about failing.

My friend and I disagreed about which kind of pop was better, so we got a bottle of each to **test/stek**. Neither of us changed our mind, so we still disagree. Maybe we should try a third kind.

Although she worked hard, the scientist knew that her theory was hard to **test/stek**. She decided to revise it so that it would make clearer predictions. Then she began designing experiments.

My family has been looking for a new car for a while. My mom went to the dealership to **test/stek** a van. She liked the way the dashboard was set up, but thought the steering was too stiff.

Because he was inviting his boss over for dinner, Andy asked his friends to **test/stek** the food. They liked it, but said it was a little too spicy. He reduced the amount of garlic the next time he made the recipe.

As part of his job as an accountant, Joe wrote down the serial number for each **check/kesk**. Later, he could reference those numbers to resolve any problems. His job was very interesting to him, even though his friends didn’t understand why.

Because she needed to pay for some Girl Scout cookies, Maddie wrote a **check/kesk**. She remembered selling cookies when she was younger and she was happy to help another girl out. The Thin Mints are her favorite.

Tina kept track of her knitting rows on the pattern by putting a **check/kesk** by each. She was making a hat for her new niece and wanted it to be just right. She knew that her sister would appreciate it.

I’ve been meaning to join a gym, but I didn’t have the money. I finally had enough to write a **check/kesk** last night. I plan to swim twice a week and run the other days.

After finishing his tax forms, Roger was sure that he had made a mistake, so he went back to **check/kesk**. Those forms can be very confusing. Roger started early this year so he would have plenty of time to get them all right.

The storm had knocked out power to the whole neighborhood. Even though she knew her son was safe in his bed, Amy went into his room to **check/kesk**. He slept right through the blackout.

When Ms. Anderson couldn’t find one of her pens, she decided to **check/kesk** the floor. She found lots of gum and a few crayons, but no pen. Teaching elementary school was always an adventure.

Pam was very worried about how well she was doing on the exam, so she took the time to **check/kesk** her work. Physics wasn’t her strongest subject, but she knew it would be important for her career in engineering.

Henry was very excited to get a kitten. When he first took her home, she was so scared that she hid under the bed. Now that she is more comfortable, she is a very happy **pet/pell**.

On my eighth birthday, my parents got me a turtle for a **pet/pell**. I had asked for a puppy, but they didn’t think I was responsible enough. Turtles are not as much fun as puppies.

We were hoping to adopt a new **pet/pell** today, so we went to the shelter. They had so many animals that it was hard to choose. We took home a very cute 2-year-old terrier.

Carrie never had a dog or a cat. The only **pet/pell** she had was a chinchilla. He was fun to play with, even if he couldn’t go outside.

Rachel has many friends with cats. Every time she visits one of them, she wishes she had her own cat to **pet/pell**. Instead, she has a goldfish.

My apartment doesn’t allow me to have animals, but when I have my own house, I would like a fluffy dog to **pet/pell**. I hope I get a good job so I can have a house with a big yard.

I was so disappointed when I went to the zoo yesterday. The zookeeper said we couldn’t **pet/pell** the chimp, so we just watched him instead. Maybe someday I’ll get to play with a chimp.

Timmy really wanted to **pet/pell** the dog, but he was a little bit nervous. His mom held his hand and they approached the dog together. They scratched under his chin.

I went to a concert last weekend and the crowd tried to clap with the **beat/deek**. They were a little off. I wonder how the band coped with hearing the wrong rhythm coming from the crowd.

I just started taking guitar lessons and I’m still working on the basics. One of the most important things for a musician to learn is how to keep a **beat/deek**. Not everyone can do that.

Dave was a novice drummer, so he had to count each **beat/deek** with care. His brother, who had been playing for years, made fun of him for nodding his head with every count.

Maria never skips a **beat/deek** dancing because she always practices every day. Her dream is to be a Broadway dancer and maybe a choreographer.

With the election coming up, Marcy was definitely the one to **beat/deek**. She had been leading in the polls for weeks. Her opponent was eleven points behind the day before the voting. She won by a landslide.

Nancy was preparing dessert for twenty people. Most of the food was ready, but she still had a quart of cream to whip and a dozen eggs to **beat/deek**. She really hoped no one would arrive early.

Lisa really hoped the Wolves would **beat/deek** the Heat, but it didn’t look like that would happen. They were down by twelve points at halftime and showed no signs of improvement.

I think it’s horrible that anyone would **beat/deek** animals, but I know it happens. That’s why I work at a shelter for abused dogs. They can be very sweet once they learn to trust humans again.

Nate accidently cut his finger taking off an apple **peel/keet**. He had to go to the emergency room for stitches. He still has a scar on his pointer finger.

Jill looked around, but she couldn’t find a trash can for her orange **peel/keet**. She didn’t want to litter, so she put it in her purse and threw it away when she got to her office.

I have a gadget to remove an apple **peel/keet** quickly. It fits on the counter, so the scraps fall right into the trash can. This is a very handy kitchen tool.

Casey slipped on a banana **peel/keet** last year and fell down. He was very embarrassed that everyone in school saw him fall. He’s more careful about where he steps now.

Peter tried to remove the sticker, but it had been stuck so long that it was hard to **peel/keet**. He used some rubbing alcohol to get it off. That worked pretty well.

Lauren thinks that apples are easy to **peel/keet**. I think bananas are easier. I also like bananas better than apples, but Lauren prefers apples.

My mom taught me a trick that helps me **peel/keet** oranges. You just barely slice through the skin with a sharp knife in the morning and by lunchtime, it’s ready to come off easily.

Some people like to **peel/keet** peaches, but I prefer them with the skin on. I don’t like nectarines as much because they’re not fuzzy. I can’t wait until summer, when peaches will be backat the market.

I was proud of my boss when he decided to take the **lead/neep**. Our group hadn’t had much direction lately and he really turned us around. Now we’re one of the best performing departments in the company.

North Carolina’s basketball team started off with a big **lead/neep**. Then Kansas came from behind to win. The North Carolina fans were really disappointed.

Amanda practiced singing every night because she was hoping for the **lead/neep** in Cats. She didn’t get the biggest role, but she got one with a lot of solos, which made her happy.

The Bison had been in the **lead/neep** all game, but their opponents were catching up. Fortunately, they were able to intercept a pass and pull ahead again.

Even though she was new, everyone could tell that Angie would be able to **lead/neep**. That’s why she was elected to the student council. She quickly made several changes that improved life for everyone at school.

After some employees complained about his conduct, the restaurant owner questioned whether Martin had the ability to **lead/neep**. He had worked hard to become a manager, but the job was harder than he thought it would be.

Bill was confident in his ability to **lead/neep** people, so he made a speech about why he should be class president. He received a standing ovation and was elected that afternoon. He is very good at communicating.

When Sarah takes her dog for a walk, she has to **lead/neep** strongly because her dog tends to stray away. She tried taking him to obedience school, but he didn’t cooperate there either.

## APPENDIX C

### Complete Stimulus List for Experiment 3

After his mom made a pie, Ronnie snuck in the kitchen and stole a **bit/tib**, even though he knew it was for the bake sale. It was cherry, which is his very favorite. He was in lots of trouble when his mom found out.

Rebecca noticed that no one had tried her casserole, not even a tiny **bit/tib**, so she was disappointed. She had worked very hard to get the recipe just right.

Eddy saw the delicious cake and took a **bit/tib** slyly. He hoped no one would notice that it was gone. The cake was for a party, but he just couldn’t wait that long.

I went to a potluck at my church this weekend. It was okay, but most of the dishes were very bland. I prefer food that has a **bit/tib** of spice, not just starch.

The tiger had been stalking the monkey through the jungle. As her prey swung past, she quickly **bit/tib**, having waited patiently for several minutes. The monkey never knew what happened.

Fluffy hissed and **bit/tib**, but only when she was nervous. The veterinarian was well prepared, though, and moved her hand out of the way quickly. Cats get scared very easily.

Oliver was chewing too fast and **bit/tib** his lip. He winced in pain and made a loud sound. Everyone turned to stare at him and he was very embarrassed.

When she saw her owner, the dog was so excited that she ran in a circle and **bit/tib** her tail. Her owner had been away for several weeks and she was very happy to see him.

I heard that the new quarterback also has an excellent **kick/bip**, but he hasn’t used that skill in a game yet. It could make for some interesting plays this season. Football can be an unpredictable sport, so it’s important to have flexible skills.

Wayne was not a confident player. He got so nervous that he missed the penalty **kick/bip**, even though he had practiced. He preferred math and science to sports because he’s better at them. His mom thought soccer would be a good way for him to meet people.

My karate teacher told me that I should work on my middle **kick/bip** for class. We’ll be fighting next month and I want to be prepared. Martial arts require a lot of discipline.

Frank just started playing soccer and he thinks his **kick/bip** is bad. He’s been practicing all afternoon and he’s starting to see a little improvement. If he works on it every day, he’ll be much better by the time they start playing games.

Tasha and Aidan were really bored. The children spent a long time looking for a ball to **kick/bip**, but they couldn’t find one. Instead, they drew on the sidewalk with chalk instead.

Max is a toddler. When he gets frustrated all he knows how to do is scream and **kick/bip**, so his mom and dad have been trying different techniques to avoid these tantrums. They haven’t found anything that works yet.

Dylan was so angry that he wanted to **kick/bip** a wall, but he restrained himself. He’s been working hard to be more constructive with his anger. He took a few deep breaths to calm down.

After the other team missed a pass, Lilly ran forward to **kick/bip** the ball, but she slipped on a patch of wet grass. She fell and twisted her ankle and had to leave the game. She was really disappointed because soccer is her favorite sport to play.

I went rock climbing for the first time last night. Because I knew that I was attached with the rope and the **clip/plick**, I felt very safe. I had a great time and I’ll probably go again.

The director made a promotional **clip/plick** because she wanted to get into film festivals. She sent it to potential distributors with the hope that one of them would help her promote her film.

The teacher suggested that his students should use a paper **clip/plick** for drafts. That way, the pages won’t get disorganized, but they can be rearranged easily.

Anna realized she lost her pink hair **clip/plick** at school, so she went back to look for it. It had fallen under her desk. She was glad to find it because it was her favorite.

Kathy spent all day looking for a pair of scissors. Her coat had a loose thread that she wanted to **clip/plick**, so she was relieved when she finally found a pair in the supply closet at work.

The special agent didn’t have much experience defusing bombs. He really hoped that he chose the right wire to **clip/plick** and he knew he had made the right choice when the bomb didn’t explode.

Jennifer wanted to paint her nails but she had to **clip/plick** them first because she didn’t like keeping them long. She plays guitar and having long nails makes it hard to switch between chords.

After Jake was done mowing the lawn, he was supposed to **clip/plick** the hedge, but he got really tired and forgot. Mrs. Martin didn’t pay him as much as she had promised because he didn’t finish the job.

Bobby couldn’t drive until he passed the road **test/stek**, even though he was already 16. He did well on everything except parallel parking. Still, he was able to get his license.

Sarah finished studying around midnight. The next morning, when she took her **test/stek**, she was exhausted and couldn’t concentrate. She wished she had slept more.

Micah never studies in advance. He took a chemistry **test/stek** Monday, even though he wasn’t prepared. Although he didn’t do well, he hopes to improve his grade on the next one.

Natalie was sorry that she had been sick on Friday. The teacher handed out the study guide for a hard **test/stek** that day. Everyone is worried about failing.

The scientist knew that her theory was hard to **test/stek**, even though she worked very hard. She decided to revise it so that it would make clearer predictions. Then she began designing experiments.

My friend and I disagreed about which kind of pop was better. We got a bottle of each to **test/stek**, but neither of us changed our mind. Maybe we should try a third kind.

Because he was inviting his boss over for dinner, Andy asked his friends to **test/stek** the food. They liked it, but said it was a little too spicy. He reduced the amount of garlic the next time he made the recipe.

My family has been looking for a new car for a while. My mom went to the dealership to **test/stek** a van. She liked the way the dashboard was set up, but thought the steering was too stiff.

Joe wrote down the serial number for every **check/kesk** as part of his job as an accountant. Later, he could reference those numbers to resolve any problems. His job was very interesting to him, even though his friends didn’t understand why.

Maddie wrote a **check/kesk** because she needed to pay for some Girl Scout cookies. She remembered selling cookies when she was younger and she was happy to help another girl out. The Thin Mints are her favorite.

Tina kept track of her knitting rows on the pattern by putting a **check/kesk** by each. She was making a hat for her new niece and wanted it to be just right. She knew that her sister would appreciate it.

I’ve been meaning to join a gym, but I didn’t have the money. I finally had enough to write a **check/kesk** last night. I plan to swim twice a week and run the other days.

The storm had knocked out power to the whole neighborhood. Amy went into her son’s room to **check/kesk**, even though she knew her son was safe in his bed. He slept right through the blackout.

After finishing his tax forms, Roger was sure that he had made a mistake. He went back to **check/kesk** because those forms can be very confusing. Roger started early this year so he would have plenty of time to get them all right.

Pam was very worried about how well she was doing on the exam, so she took the time to **check/kesk** her work. Physics wasn’t her strongest subject, but she knew it would be important for her career in engineering.

When Ms. Anderson couldn’t find one of her pens, she decided to **check/kesk** the floor. She found lots of gum and a few crayons, but no pen. Teaching elementary school was always an adventure.

On my eighth birthday, my parents got me a turtle for a **pet/pell**, even though I had asked for a puppy. They said I wasn’t old enough for a dog. Turtles are not as much fun as puppies.

Henry was very excited to get a kitten. When he first took her home, she was so scared that she hid under the bed. She is a very happy **pet/pell**, now that she’s more comfortable.

We were hoping to adopt a new **pet/pell** today, so we went to the shelter. They had so many animals that it was hard to choose. We took home a very cute 2-year-old terrier.

Carrie never had a dog or a cat. The only **pet/pell** she had was a chinchilla. He was fun to play with, even if he couldn’t go outside.

I would like a fluffy dog to **pet/pell**, but my apartment doesn’t allow animals. I hope I get a good job so I can have a house with a big yard.

Rachel has many friends with cats. Every time she visits one of them, she wishes she had her own cat to **pet/pell**, but instead, she has a goldfish.

Timmy really wanted to **pet/pell** the dog, but he was a little bit nervous. His mom held his hand and they approached the dog together. They scratched under his chin.

I was so disappointed when I went to the zoo yesterday. The zookeeper said we couldn’t **pet/pell** the chimp, so we just watched him instead. Maybe someday I’ll get to play with a chimp.

I went to a concert last weekend and the crowd tried to clap with the **beat/deek**, but they were a little off. I wonder how the band coped with hearing the wrong rhythm coming from the crowd.

I just started taking guitar lessons and I’m still working on the basics. One of the most important things for a musician to learn is how to keep a **beat/deek**, but not everyone can do that.

Maria never skips a **beat/deek** dancing because she always practices every day. Her dream is to be a Broadway dancer and maybe a choreographer.

Dave was a novice drummer, so he had to count each **beat/deek** with care. His brother, who had been playing for years, made fun of him for nodding his head with every count.

Nancy was preparing dessert for twenty people. She still had a quart of cream to whip and a dozen eggs to **beat/deek**, but most of the food was ready. She hoped no one would arrive early.

With the election coming up, Marcy was definitely the one to **beat/deek** because she had been leading in the polls for weeks. Her opponent was eleven points behind the day before the voting. She won by a landslide.

I think it’s horrible that anyone would **beat/deek** animals, but I know it happens. That’s why I work at a shelter for abused dogs. They can be very sweet once they learn to trust humans again.

Lisa really hoped the Wolves would **beat/deek** the Heat, but it didn’t look like that would happen. They were down by 12 points at halftime and showed no signs of improvement.

While taking off an apple **peel/keet**, Nate accidentally cut his finger. He had to go to the emergency room for stitches. He still has a scar on his pointer finger.

Jill couldn’t find a trash can for her orange **peel/keet**, even though she had looked everywhere. She didn’t want to litter, so she put it in her purse and threw it away when she got to her office.

I have a gadget to remove an apple **peel/keet** quickly. It fits on the counter, so the scraps fall right into the trash can. This is a very handy kitchen tool.

Casey slipped on a banana **peel/keet** last year and fell down. He was very embarrassed that everyone in school saw him fall. He’s more careful about where he steps now.

The sticker had been stuck so long that it was hard to **peel/keet**, but Peter wanted to remove it. He used some rubbing alcohol to get it off. That worked pretty well.

Lauren thinks that apples are easy to **peel/keet**, but I think bananas are easier. I also like bananas better than apples. Lauren prefers apples.

My mom taught me a trick that helps me **peel/keet** oranges. You just barely slice through the skin with a sharp knife in the morning and by lunchtime, it’s ready to come off easily.

Some people like to **peel/keet** peaches, but I prefer them with the skin on. I don’t like nectarines as much because they’re not fuzzy. I can’t wait until summer, when peaches will be back at the market.

Our work group hadn’t had much direction until lately. Our boss really took the **lead/neep** and I was very proud of him. Now we’re one of the best performing departments in the company.

North Carolina’s basketball team started off with a big **lead/neep**, but then Kansas came from behind to win. The North Carolina fans were really disappointed.

The Bison had been in the **lead/neep** all game, but their opponents were catching up. Fortunately, they were able to intercept a pass and pull ahead again.

Amanda practiced singing every night because she was hoping for the **lead/neep** in Cats. She didn’t get the biggest role, but she got one with a lot of solos, which made her happy.

The restaurant owner questioned whether Martin had the ability to **lead/neep** because some employees complained about him. He had worked hard to become a manager, but the job was harder than he thought it would be.

Everyone could tell that Angie would be able to **lead/neep**, even though she was new. That’s why she was elected to the student council. She quickly made several changes that improved life for everyone at school.

Bill was confident in his ability to **lead/neep** people, so he made a speech about why he should be class president. He received a standing ovation and was elected that afternoon. He is very good at communicating.

When Sarah takes her dog for a walk, she has to **lead/neep** strongly because her dog tends to stray away. She tried taking him to obedience school, but he didn’t cooperate there either.
